# Vaccination and Infection as Causative Factors in Japanese Patients With Rasmussen Syndrome: Molecular Mimicry and HLA Class I

**DOI:** 10.1080/17402520600589522

**Published:** 2006

**Authors:** Yukitoshi Takahashi, Kazumi Matsuda, Yuko Kubota, Jiro Shimomura, Etsuko Yamasaki, Tatsuya Kudo, Katsuyuki Fukushima, Hitoshi Osaka, Noriyuki Akasaka, Atsushi Imamura, Shinji Yamada, Naomi Kondo, Tateki Fujiwara

**Affiliations:** 1 National Epilepsy Center Shizuoka Institute of Epilepsy and Neurological Disorders Shizuoka 420-868 Japan; 2 Kanagawa Children's Medical Center Mutsukawa 2-138-4, Minami-ku Yokohama 232-8555 Japan; 3 National Nishi-Niigata Central Hospital 1-14-1 Masago Niigata 950-2085 Japan; 4 Gifu Prefectural Gifu Hospital 4-6-1 Noishiki Gifu 500-8717 Japan; 5 Chunou Kosei Hospital 5-1 Wakakusa-dori Seki 501-3802 Japan; 6 Department of Pediatrics Gifu University School of Medicine Gifu 500-8705 Japan

## Abstract

Rasmussen syndrome is an intractable epilepsy with a putative causal relation with cellular and humoral autoimmunity. Almost half of the patients have some preceding causative factors, with infections found in 38.2%, vaccinations in 5.9% and head trauma in 8.9% of Japanese patients. In a patient with seizure onset after influenza A infections, cross-reaction of the patient's lymphocytes with GluRε2 and influenza vaccine components was demonstrated by lymphocyte stimulation test. Database analyses revealed that influenza A virus hemagglutinin and GluRε2 molecules contain peptides with the patient's HLA class I binding motif (HLA − A*0201). The relative risks of HLA class I genotypes for Rasmussen syndrome are 6.1 (A*2402), 6.4 (A*0201), 6.3 (A*2601) and 11.4 (B*4601). The relative risks of HLA class I-A and B haplotypes are infinity (A*2601+B*5401), 21.1 (A*2402+B*1501), 13.3 (A*2402+B*4801) and 5.1 (A*2402+B*5201). Some alleles and haplotypes of HLA class I may be the risk factors in Japanese patients. Cross-reactivity of cytotoxic T lymphocytes may contribute to the processes leading from infection to the involvement of CNS.


*Clinical & Developmental Immunology*, June–December 2006; 13(2–4): 381–387

Taylor & Francis

Taylor & Francis Group

### Vaccination and infection as causative factors in Japanese patients with Rasmussen syndrome: Molecular mimicry and HLA class I

YUKITOSHI TAKAHASHI[1], KAZUMI MATSUDA[1], YUKO KUBOTA[1], JIRO SHIMOMURA[1], ETSUKO YAMASAKI[1], TATSUYA KUDO[1], KATSUYUKI FUKUSHIMA[1], HITOSHI OSAKA[2], NORIYUKI AKASAKA[3], ATSUSHI IMAMURA[4], SHINJI YAMADA[5], NAOMI KONDO[6] & TATEKI FUJIWARA[1]

[1]*National Epilepsy Center, Shizuoka Institute of Epilepsy and Neurological Disorders, 886 Urushiyama Aoi-ku, Shizuoka 420-8688, Japan,* [2]*Kanagawa Children's Medical Center, Mutsukawa 2-138-4, Minami-ku, Yokohama 232-8555, Japan,* [3]*National Nishi-Niigata Central Hospital, 1-14-1 Masago, Niigata 950-2085, Japan,* [4]*Gifu Prefectural Gifu Hospital, 4-6-1 Noishiki, Gifu 500-8717, Japan,* [5]*Chunou Kosei Hospital, 5-1 Wakakusa-dori, Seki 501-3802, Japan, and* [6]*Department of Pediatrics, Gifu University School of Medicine, Gifu 500-8705, Japan*

**Abstract**

Rasmussen syndrome is an intractable epilepsy with a putative causal relation with cellular and humoral autoimmunity. Almost half of the patients have some preceding causative factors, with infections found in 38.2%, vaccinations in 5.9% and head trauma in 8.9% of Japanese patients. In a patient with seizure onset after influenza A infections, cross-reaction of the patient's lymphocytes with GluRε2 and influenza vaccine components was demonstrated by lymphocyte stimulation test. Database analyses revealed that influenza A virus hemagglutinin and GluRε2 molecules contain peptides with the patient's HLA class I binding motif (HLA − A*0201). The relative risks of HLA class I genotypes for Rasmussen syndrome are 6.1 (A*2402), 6.4 (A*0201), 6.3 (A*2601) and 11.4 (B*4601). The relative risks of HLA class I-A and B haplotypes are infinity (A*2601 + B*5401), 21.1 (A*2402 + B*1501), 13.3 (A*2402 + B*4801) and 5.1 (A*2402 + B*5201). Some alleles and haplotypes of HLA class I may be the risk factors in Japanese patients. Cross-reactivity of cytotoxic T lymphocytes may contribute to the processes leading from infection to the involvement of CNS.

**Keywords:** *Rasmussen syndrome, HLA, cytotoxic T cells, influenza, vaccination, epilepsy*

### Introduction

Rasmussen's encephalitis is a slowly progressive, autoimmune-mediated chronic inflammatory disease of the CNS. The mean age of onset is 7.4 years. The disease may be preceded by some causative factors including infection, and the initial seizure episode manifests various forms such as partial onset generalized tonic-clinic (pGTC) seizures (30%), focal motor seizures (26%) and complex partial seizures (CPS) (26%) (Andermann 1991). One third of the patients have preceding infections within 1 month before onset. Patients with typical Rasmussen's encephalitis manifest frequent intractable partial motor seizures in the acute phase, characteristically epilepsia partialis continua (EPC) (56%). Patients begin to manifest EPC 1.8 years after the onset of epilepsy, but the seizure frequency decreases markedly in the residual stage (Andermann 1991, Bien et al. 2002b) (Figure 1). Patients in the residual stage are affected by hemiplegia (96%), mental deterioration (85%), visual field defect (49%), and cortical sensory defect (29%) (Andermann 1991). Histological examination reveals infiltration of T lymphocytes and microglia cells, astrocytosis, and neuronal loss in the lesion (Aguilar and Rasmussen 1960, Andermann 1991, Farrell et al. 1995). Functional hemispherectomy is the only reliable therapy when the non-dominant side is involved, but hemiparesis and hemianopsia are

Correspondence: Y. Takahashi, Shizuoka Institute of Epilepsy and Neurological Disorders, National Epilepsy Center, 886 Urushiyama Aoi-ku, Shizuoka 420-8688, Japan. Tel: 81 54 245 5446. Fax: 81 54 247 9781. E-mail: takahashi-ped@umin.ac.jp

ISSN 1740-2522 print/ISSN 1740-2530 online © 2006 Taylor & Francis
DOI: 10.1080/17402520600589522

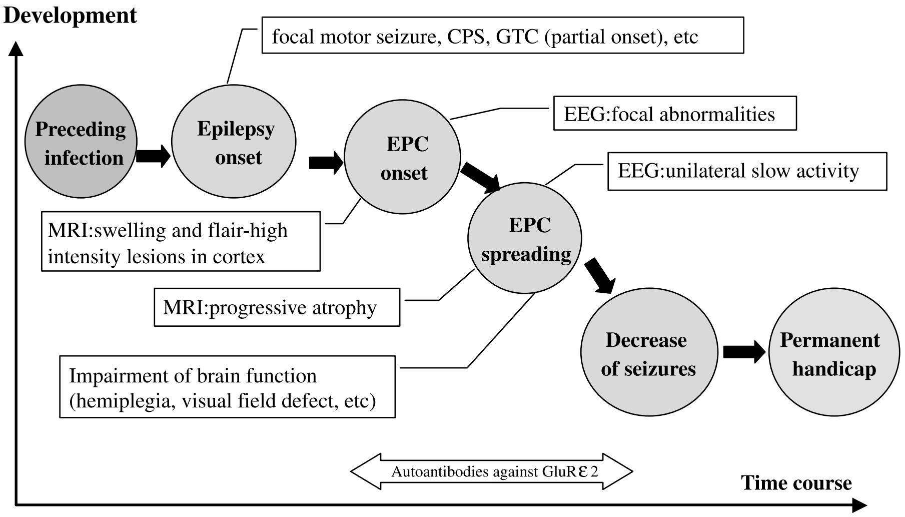

Figure 1. Schematic presentation of the typical clinical course of Rasmussen syndrome. After preceding infections, epileptic seizures (focal motor seizures or partial onset generalized tonic clonic convulsions, etc.) appear, followed by progressive deterioration of clinical symptoms and aggravation of EEGs and MRI abnormalities. In the residual stage, permanent handicap is observed, but epileptic seizures decrease. GTC, generalized tonic clonic seizure; GluR, glutamate receptor; EPC, epilepsia partialis continua.

unavoidable after operation. When the dominant side is affected, there is no effective therapy.

Viral infections were implicated as the causal agent of Rasmussen's encephalitis in early investigations (Andermann 1991), and direct infection by several candidate viruses (CMV, tick-borne encephalitis virus, etc.) has been postulated as one of the possible mechanisms causing the disease (Takahashi 2006) (Figure 2).

Rogers et al. (1994) reported glutamate receptor 3 (GluR3) as an autoantigen in Rasmussen's encephalitis, and proposed autoantibodies against this molecule to be one cause of Rasmussen's encephalitis (humoral autoimmune hypothesis). Their report

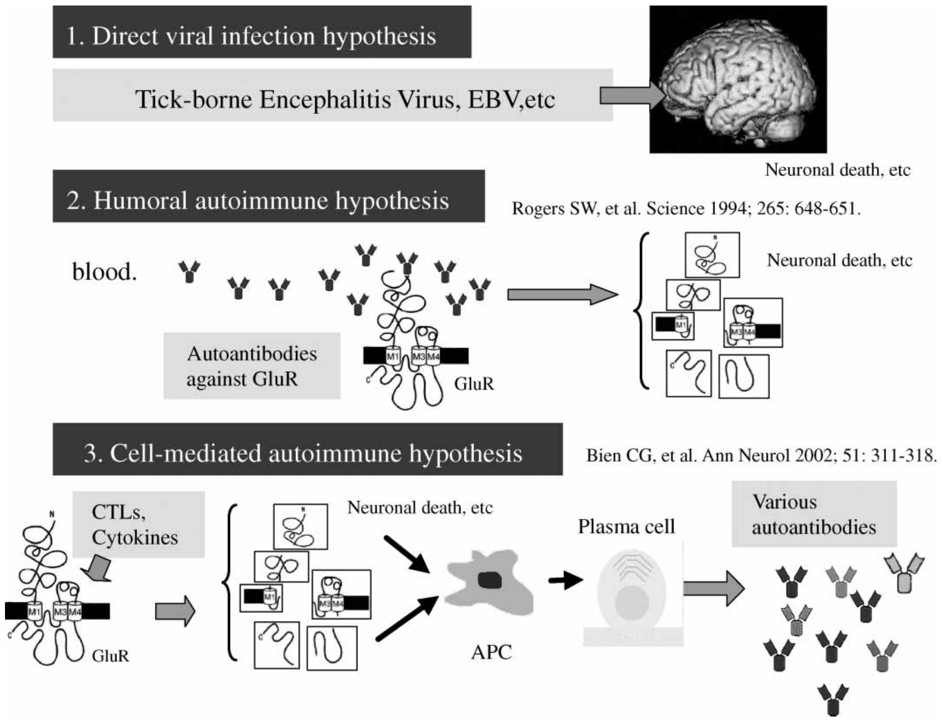

Figure 2. Hypotheses of autoimmune pathologies in Rasmussen syndrome. Direct viral infection hypothesis presumes that viral particles invade the brain and cause neuronal death, etc. Humoral autoimmune hypothesis supposes that autoantibodies against neural molecules (GluR3, etc.) play the primary roles to develop Rasmussen syndrome. Cell-mediated autoimmune hypothesis speculates that CTLs and/or cytokines from $CD4^{+}$T cells play the primary roles. EBV, Ebstein bar virus; GluR, glutamate receptor; APC, antigen-presenting cells.

introduced a new perspective of autoimmune-mediated mechanism to the field of epilepsy. Several pathological roles of autoantibodies against GluR3 have been demonstrated, including excitotoxicity (Levite and Hermelin 1999), complement-dependent cell death (He et al. 1998) and membrane attack complex (MAC) (Xiong and McNamara 2002, Xiong et al. 2003) have been shown, although induction of currents through GluR remains controversial (Twyman et al. 1995, Watson et al. 2004). The MAC is composed of several complements, and appears to induce functional pore in cell membrane, leading to depolarization and osmotic lysis of neurons. These data indicate that autoantibodies can directly cause impairment of neural functions.

On the other hand, Bien et al. (2002a) proposed the destruction of neurons by cytotoxic T cells (CTLs) as a new pathogenic mechanism in Rasmussen's encephalitis (cell-mediated autoimmune hypothesis). Lymphocytic infiltration containing predominantly T cells and sparsely B cells can be observed in surgically resected tissues from patients with Rasmussen's encephalitis (Farrell et al. 1995), and local CNS immune responses in Rasmussen's encephalitis include clonal expansion of T cells responding to discrete antigen epitopes (Li et al. 1997). Peripheral blood lymphocytes from patients are sensitized to GluRε2 (Takahashi et al. 2005). Heterogeneous autoantibodies against neuronal molecules (including GluR3, GluRε2, neuronal acetylcholine receptor alpha7, and munc-18) (Yang et al. 2000, Watson et al. 2001, Takahashi et al. 2003) and glial cells (Roubertie et al. 2005) are detected in Rasmussen syndrome. Autoantibodies against GluRε2 have epitopes predominantly in intracellular domains, and show epitope spreading evolutionally (Takahashi et al. 2003). We postulated that the autoimmune-mediated mechanism for the development of Rasmussen syndrome involves primarily cellular autoimmunity mediated by cytotoxic T cells, and evolutionarily involves humoral autoimmunity mediated by autoantibodies (Takahashi et al. 2003, 2005). These autoimmune mechanisms of epileptogenesis after infections can be classified as parainfectious mechanisms (Figure 3) (Takahashi 2006).

### Causative factors of in patients with Rasmussen syndrome

In our epilepsy center, 44% of Japanese patients with Rasmussen syndrome had prior infections or vaccinations, and approximately 8% had head trauma as preceding causative factors, and the frequencies are almost same in patients with EPC and in those without EPC (Table I) (Takahashi 2006). The microbes causing infections were not identified in the majority of patients, except in three patients infected by influenza virus and one patient by mycoplasma. Likewise, in the study conducted at Montreal Neurological Institute, the causative microbes were not documented except measles (encephalitis) and varicella (Andermann 1991).

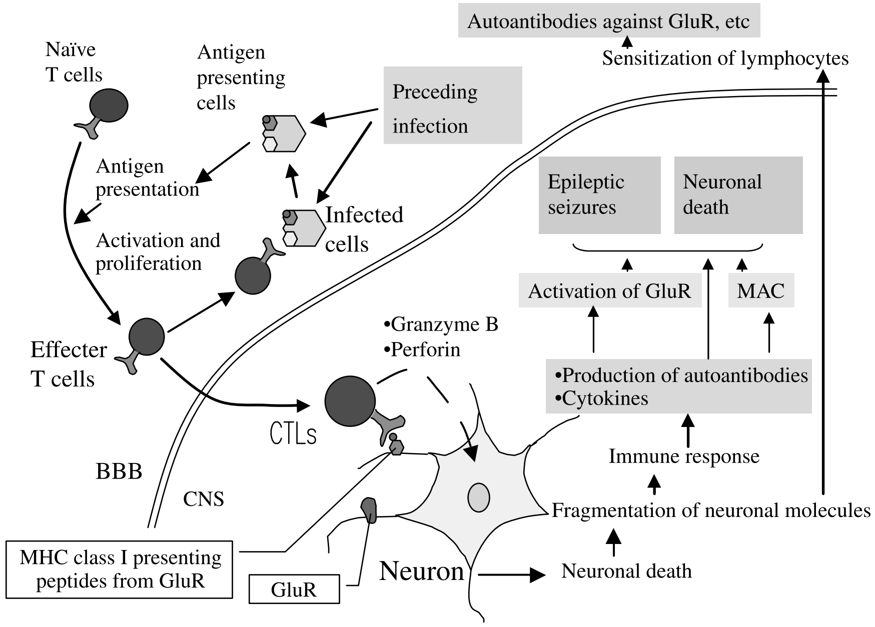

Figure 3. Involvements of CTLs and autoantibodies in the hypothetic mechanisms of the development of Rasmussen syndrome. Effecter T cells activated by preceding infections or vaccinations reach the CNS by crossing blood brain barrier, and cross-react with neurons, etc. resulting in apoptosis. Neuronal death leads to production of autoantibodies against CNS molecules and cytokines, which might contribute to the further neuronal death or epileptogenesis. Fragmented neuronal molecules reach systemic circulation and sensitize lymphocytes, resulting in production of autoantibodies in the blood. BBB, blood brain barrier; CNS, central nervous system; CTLs, cytotoxic T cells; GluR, glutamate receptor; MAC, membrane attack complex.

Table I. Causative factors in 34 patients with Rasmussen syndrome.

| | EPC type | Non-EPC type | Total |
|---|---|---|---|
| Age of onset | 6.3 ± 5.6 | 8.7 ± 8.0 | 7.4 ± 6.7 |
| Preceding infections | 8 (40.0%) | 5 (35.7%) | 13 (38.2%) |
| Fever only | 4 | 1 | 5 |
| Upper respiratory infection | 2 | 1 | 3 |
| Influenza | 1 | 2 | 3 |
| Mycoplasma | 0 | 1 | 1 |
| Aseptic meningitis | 1 | 0 | 1 |
| Vaccination | 1 (5.0%) | 1 (7.1%) | 2 (5.9%) |
| Head trauma | 2 (10.0%) | 1 (7.1%) | 3 (8.8%) |
| None | 9 (45.0%) | 7(50.0%) | 16(47.1%) |
| Total | 20 | 14 | 34 |

Therefore, it remains unknown whether specific microbes are involved in the development of Rasmussen syndrome. The contribution of molecular mimicry between microbial and neuronal molecules and degeneracy of T cell receptor recognition to the development of Rasmussen syndrome (autoimmune-mediated epilepsies) will be discussed later.

We encountered two patients who developed Rasmussen syndrome after vaccination, although vaccination was not reported as a causative factor in the series of Montreal Neurological Institute. One patient had EPC type Rasmussen syndrome. This patient received Japanese encephalitis vaccination at the age of 15 years. Two months after vaccination, he had the initial epileptic seizure, and subsequently evolved to intractable and frequent complex partial seizures (CPSs) and EPC. MRI lesions in left frontal lobe and autoantibodies against GluRε2 were detected. Focal resection of the left frontal lobe failed to control epileptic seizures, psychiatric symptoms and deterioration. Another patient had non-EPC type Rasmussen syndrome. This patient received measles–mumps–rubella triple vaccine at the age of 1 year. Three weeks later, he was affected by aseptic meningitis caused by the vaccination. He had intractable epileptic seizures from the age of two, and psychiatric symptoms (including anxiety) evolved subsequently. At the age of 14 years, right frontal lobectomy was conducted and successfully controlled the seizures.

Head trauma also was not reported as a causative factor in the patients of Montreal Neurological Institute, but we identified three patients with Rasmussen syndrome possibly related to preceding head trauma. As we sometimes experience patients with aseptic meningitis after head trauma, head trauma may facilitate the invasion of inflammatory T cells into the CNS. In patients with post-concussion syndrome after mild head injury, focal cortical dysfunction may occur in conjunction with the disruption of the blood brain barrier (Korn et al. 2005).

In the following sections, infection as a causative factor in Rasmussen syndrome will be illustrated using a specific case in which Rasmussen syndrome developed after influenza infection, especially focusing on the roles of molecular mimicry and HLA class I.

### A case of Rasmussen syndrome after influenza A infection, and cross-reaction of lymphocytes

This patient, a boy, was 8 year-old at the time of this report (Case 1). His family history was unremarkable, with ovarian cyst in his mother and parkinsonism in his maternal grandfather. At the age of 3 years and 11 months, he had febrile generalized convulsion following an episode of influenza A infection that was confirmed by antigen detection from a nasal sample. Before the influenza infection, he had no neurological symptoms and no other preceding conditions that might precipitate the convulsions. Soon after the initial convulsion, at age 4, he had febrile convulsive status of left extremities associated with repeated influenza A infection. CSF was normal, and CT revealed no abnormalities. At the age of 4 years and 1 month, afebrile convulsive status appeared and phenobarbital (50 mg) was prescribed. Thereafter, his seizures became progressively intractable, in spite of a combination of several antiepileptic drugs (carbamazepine, zonisamide, valproic acid and phenytoin). At the age of 5 years and 4 months, EPC appeared after phenytoin was stopped abruptly and clobazam was added. He was referred to our epilepsy center for the treatment of Rasmussen syndrome at the age of 5 years and 5 months. He had hemiparesis of left extremities, EPC of left lower extremity, and several partial seizures in a day (Figure 4). After functional hemispherectomy, seizures were controlled completely. At age eight, he walked to school and attended a normal elementary school.

At presentation, IgG-autoantibodies against GluRε2 were detected in serum and CSF samples (Takahashi et al. 2003), but IgM-autoantibody was negative. The stimulation index obtained in the lymphocyte stimulation test (LST) stimulated with homogenates containing GluRε2 ($^{3}$H-thymidine

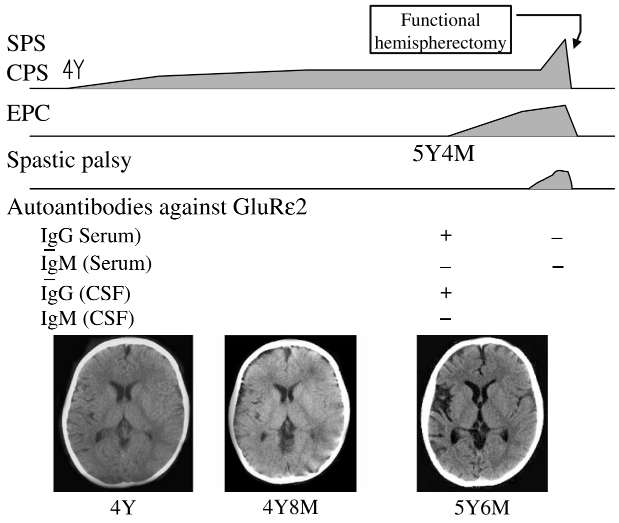

Figure 4. Clinical course of Case 1. Epilepsy occurred at the age of four, and progressive atrophy of right hemisphere started at the age of 5 year and 6 months. IgG-autoantibodies against GluRε2 were positive on admission, but became negative after functional hemispherectomy that successfully controlled seizures. SPS, simple partial seizure; CPS, complex partial seizure; EPC, epilepsia partialis continua.

uptake with stimulation/control $^{3}$H-thymidine uptake) was 2.78 (Takahashi et al. 2005), and was higher than normal controls (0.63, 1.67) tested simultaneously (Figure 5). When LST was conducted by co-stimulation with homogenates containing GluRε2 and influenza vaccine, the stimulation index was 9.19 in this patient (Case 1 in Figure 5), and was

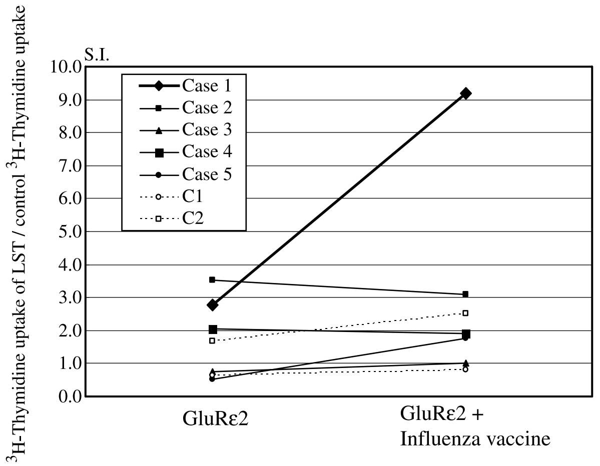

Figure 5. Stimulation index in lymphocyte stimulation test. Data on the left show stimulation indices (SI) ($^{3}$H-thymidine uptake with stimulation/control $^{3}$H-thymidine uptake) obtained in lymphocyte stimulation test (LST) stimulated with homogenate containing GluRε2 (Takahashi et al. 2005). Data on the right show SI when LST was conducted by co-stimulation with homogenates containing GluRε2 and influenza vaccine. Case 1 is the case presented in the text, with influenza A infection as a causative factor. Case 2 is Rasmussen syndrome with no causal relationship with influenza. Cases 3–5 are epileptic cases (no Rasmussen syndrome) with autoantibodies against GluRε2. C1 and C2 are normal controls.

higher compared with other patients with Rasmussen syndrome or other epilepsies not related to preceding influenza infection (Cases 2–5). A synergistic increase in stimulation index with co-stimulation (GluRε2 + influenza vaccine) compared to GluRε2 stimulation alone was observed only in the present case. These data suggest that the lymphocytes of this patient are sensitized not only by influenza antigen but also by GluRε2, and that the T cell receptors of this patient can cross-react with peptides from influenza and GluRε2.

After the T cell receptors on CTLs recognize both the HLA class I molecule and its binding peptide expressed on antigen presenting cells (APCs), these CTLs are activated into cytotoxic effector cells that are capable of invading the CNS. If through molecular mimicry and T cell receptor redundancy, the CTLs activated by microbial peptides are able to recognize the HLA class I and binding peptide expressed on neurons, then the infection-activated CTLs may induce apoptosis of neurons. Therefore, HLA class I is one of the key molecules that determines autoimmune mechanisms underlying the process leading from infection or vaccination to Rasmussen syndrome.

### HLA class I and theoretical cross-reaction of CTLs in Case 1

In the case presented above (Case 1), HLA genotyping identified HLA − A*0201, A*2402, and B*3501 (homo). HLA − A*0201 binds peptides with the following motif: [LM] − $x(3)$ − V − $x(2)$ − [VL] ($x$, free amino acid; L, leucine; M, methionine; V, valine). Database analyses (Genome Net: http://www.genome.jp/) revealed this motif in various viral molecules and neural molecules (Figure 6). If patients with HLA − A*0201 are infected by influenza A virus, the peptide *L*AIM*V*AG*L* from the hemagglutinin of influenza A is able to bind with A*0201 expressed on APCs. CTLs with T cell receptors that recognize A*0201 and the hemagglutinin peptide (influenza A) on APCs become activated and become effector CTLs. Theoretically, these effector CTLs can invade the CNS, and react with neurons expressing HLA − A*0201 and peptides containing the [LM] − $x(3)$ − V − $x(2)$ − [VL] motif, due to molecular mimicry and degeneracy of T cell receptor recognition (Uemura et al. 2003). Peptides having the HLA − A*0202 binding motif are found in various neuronal molecules, such as GluRε2 (*L*VLA*V*LA*V*, *M*LLI*V*SA*V*) and GluRε1 (*L*PLD*V*NV*V*). Therefore, we hypothesize that CTLs activated by influenza may cross-react with neurons that express GluRε2 under specific conditions such as the presence of costimulators. This hypothetic cross-reaction based on molecular mimicry of HLA-binding motif is compatible with our data of

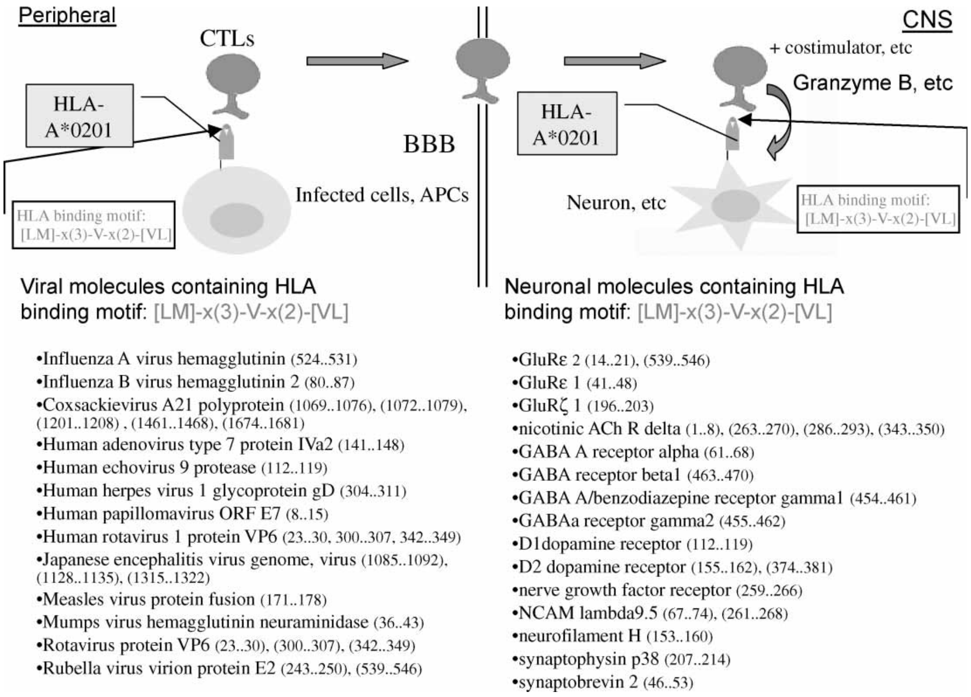

Figure 6. Viral and neural molecules containing specific binding motif for HLA − A*0201. Viral infection of host cells leads to expression of some parts of the viral peptides by binding with HLA class I molecule on the infected cells. CTLs expressing T cell receptors that recognize the peptide and HLA class I are activated, resulting in generation of effecter T cells. In individuals with HLA − A*0201, the binding motif is [LM] − $x$(3) − V − $x$(2) − [VL] ($x$, free amino acid; L, leucine; M, methionine; V, valine). Database analyses (Genome Net, http://www.genome.jp/) revealed many viral molecules containing this motif, as shown in the left column. The numbers in parentheses are sequence numbers indicating the sites of the motif. Effecter T cells are able to cross the blood brain barrier to reach the CNS, and theoretically can cross-react with neurons expressing the same motif on HLA class I, under specific conditions such as the presence of costimulator. Database analyses revealed many neural molecules including NMDA-GluRs, which contain the motif, as shown in the right column.

lymphocyte cross-reactivity between GluRε2 and influenza vaccine observed in Case 1 (Figure 5). Since CTLs activated by influenza can react with a broad spectrum of neuronal molecules, theoretically, apoptotic lesions caused by CTLs may distribute widely in the brain. On the other hand, possible interactions of the activated CTLs of Case 1 with a variety of microbial molecules (Figure 6) may explain the symptomatic aggravation triggered by infections other than influenza after the onset of Rasmussen syndrome. T cell clones from type 1 diabetes patients have been shown to react with several kinds of microbial mimicry peptides (Uemura et al. 2003). Similar molecular mimicry may also exists for HLA − A*2402 and B*3501. These molecules also have specific binding motifs. Database search identified diverse peptides containing these specific motifs in both microbial and neural molecules.

### HLA class I in patients with Rasmussen syndrome

We studied the genotypes of HLA class I in 16 Japanese patients with Rasmussen syndrome (EPC type, 9; non-EPC type, 7) by PCR amplifications. The data were analyzed statistically using Chi-square for independence test. HLA − A*2402 is a popular genotype (36.5% of Japanese population) and was found in 77.8% of EPC type patients ($p = 0.016$). The frequencies of HLA − A*0201 and HLA − A*2601 were higher in non-EPC type patients (both 42.9%) than in Japanese population (10.7 and 11.3%, respectively) ($p = 0.033$ and 0.038, respectively). HLA − B*5201was found more frequently in EPC type patients (33.3%) than in Japanese population (10.9%) ($p = 0.070$), while HLA − B*4601 was more frequent in non-EPC type patients (28.6%) than in Japanese population (3.4%) ($p = 0.025$). The relative risks of various HLA class I genotypes for Rasmussen syndrome range from 6 to 11 (Table II), which are at the same levels as systemic lupus erythematosus (DR2) and acute anterior uveitis (B27) (Marsh et al. 2000). The relative risks of HLA class I-A and B haplotypes range from 5 to ∞. Since the haplotype of A*2601 + B*5401 was not observed in 561 Japanese subjects, the risk is infinity.

The HLA class I types that have higher relative risks may have a greater potential to induce cross-reactions of CTLs between microbes and neurons, and consequently may be found at higher frequencies in patients with Rasmussen syndrome. The binding motifs of these HLA class I types probably exist frequently in molecules from microbes commonly found in Japan and in molecules derived from neurons.

Table II. HLA class I genotypes and relative risks in patients with Rasmussen syndrome.

| HLA genotypes | Clinical phenotype | Relative risk |
|---|---|---|
| HLA class I-A | | |
| A*2402 | EPC type | 6.1 |
| A*0201 | Non-EPC type | 6.4 |
| A*2601 | Non-EPC type | 6.3 |
| HLA class I-B | | |
| B*4601 | Non-EPC type | 11.4 |
| HLA − A + B haplotypes | | |
| A*2402 + B*4801 | EPC type | 13.3 |
| A*2402 + B*1501 | EPC type | 21.1 |
| A*2402 + B*5201 | EPC type | 5.1 |
| A*2601 + B*5401 | Non-EPC type | ∞ |

∞, infinity.

### Acknowledgements

The author thanks Masayoshi Mishina and Hisashi Mori for their helpful comments, and Shigeko Nishimura and Hisano Tsunogae for their skillful assistance. This study was funded in part by Research Grants (16A-3) for Nervous and Mental Disorders from the Ministry of Health, Labor and Welfare, grants-in-aid for Scientific Research I No. 15591151, 16590859, and 17591133, Health and Labour Sciences Research Grants for Research on Psychiatry and Neurological Diseases and Mental Health (H17-017) and Research on Children and Families (H16-016), and grants from The Japan Epilepsy Research Foundation.

### References

Aguilar MJ, Rasmussen T. 1960. Role of encephalitis in pathogenesis of epilepsy. AMA Arch Neurol 2:663–676.

Andermann F, editor. 1991. Chronic encephalitis and epilepsy: Rasmussen's syndrome. Boston: Butterworth-Heinemann.

Bien CG, Bauer J, Deckwerth TL, et al. 2002a. Destruction of neurons by cytotoxic T cells: A new pathogenic mechanism in Rasmussen's encephalitis. Ann Neurol 51:311–318.

Bien CG, Widman G, Urbach H, et al. 2002b. The natural history of Rasmussen's encephalitis. Brain 125:1751–1759.

Farrell MA, Droogan O, Secor DL, Poukens V, Quinn B, Vinters HV. 1995. Chronic encephalitis associated with epilepsy: Immunohistochemical and ultrastructural studies. Acta Neuropathol 89:313–321.

He XP, Patel M, Whitney KD, Janumpalli S, Tenner A, McNamara JO. 1998. Glutamate receptor GluR3 antibodies and death of cortical cells. Neuron 20:153–163.

Korn A, Golan H, Melamed I, Pascual-Marqui R, Friedman AJ. 2005. Focal cortical dysfunction and blood–brain barrier disruption in patients with Postconcussion syndrome. Clin Neurophysiol 22:1–9.

Levite M, Hermelin A. 1999. Autoimmunity to the glutamate receptor in mice—a model for Rasmussen's encephalitis? J Autoimmun 13:73–82.

Li Y, Uccelli A, Laxer KD, et al. 1997. Local-clonal expansion of infiltrating T lymphocytes in chronic encephalitis of Rasmussen. J Immunol 158:1428–1437.

Marsh SGE, Parham P, Barber LD. 2000. The HLA factsBook. London: Academic Press. p 79–83.

Rogers SW, Andrews PI, Gahring LC, et al. 1994. Autoantibodies to glutamate receptor GluR3 in Rasmussen's encephalitis. Science 265:648–651.

Roubertie A, Boukhaddaoui H, Sieso V, et al. 2005. Antiglial cell autoantibodies and childhood epilepsy: A case report. Epilepsia 46:1308–1312.

Takahashi Y. 2006. Vaccination and infection as causative factors of epilepsy. Future Neurol (in press).

Takahashi Y, Mori H, Mishina M, et al. 2003. Autoantibodies to NMDA receptor in patients with chronic forms of epilepsia partialis continua. Neurology 61:891–896.

Takahashi Y, Mori H, Mishina M, et al. 2005. Autoantibodies and cell-mediated autoimmunity to NMDA-type GluRε2 in patients with Rasmussen's encephalitis and chronic progressive epilepsia partialis continua. Epilepsia 46(Suppl 5):152–158.

Twyman RE, Gahring LC, Spiess J, Rogers SW. 1995. Glutamate receptor antibodies activate a subset of receptors and reveal an agonist binding site. Neuron 14:755–762.

Uemura Y, Senju S, Maenaka K, et al. 2003. Systematic analysis of the combinatorial nature of epitopes recognized by TCR leads to identification of mimicry epitopes for glutamic acid decarboxylase 65-specific TCRs. J Immunol 170:947–960.

Watson R, Jiang Y, Bermudez I, et al. 2004. Absence of antibodies to glutamate receptor type 3 (GluR3) in Rasmussen encephalitis. Neurology 63:43–50.

Watson R, Lang B, Bermudez I, et al. 2001. Autoantibodies in Rasmussen's encephalitis. J Neuroimmunol 118:148.

Xiong ZO, McNamara JO. 2002. Fleeting activation of ionotropic glutamate receptors sensitizes cortical neurons to complement attack. Neuron 36:363–374.

Xiong ZO, Qian W, Suzuki K, McNamara JO. 2003. Formation of complement membrane attack complex in mammalian cerebral cortex evokes seizures and neurodegeneration. J Neurosci 23: 955–960.

Yang R, Puranam RS, Butler LS, et al. 2000. Autoimmunity to munc-18 in Rasmussen's encephalitis. Neuron 28:375–383.

